# The Influence of Differences in Solvents and Concentration on the Efficacy of Propofol at Induction of Anesthesia

**DOI:** 10.1155/2016/9178523

**Published:** 2016-01-21

**Authors:** Yukako Obata, Yushi U. Adachi, Katsumi Suzuki, Taiga Itagaki, Hiromi Kato, Maiko Satomoto, Yoshiki Nakajima

**Affiliations:** ^1^Department of Intensive Care Unit, Hamamatsu University School of Medicine, 1-20-1 Handayama, Higashi-ku, Hamamatsu, Shizuoka 4313192, Japan; ^2^Department of Intensive Care Medicine, Tokyo Medical and Dental University Medical Hospital, Tokyo, Japan; ^3^Department of Anesthesia, Enshu Hospital, Hamamatsu, Japan; ^4^Pulmonary and Critical Care Medicine, Massachusetts General Hospital, Boston, USA; ^5^Department of Anesthesiology, Graduate School of Medical and Dental Sciences, Tokyo Medical and Dental University, Japan

## Abstract

*Background*. Propofol is a popular intravenous anesthetic and varieties of formulations were produced from different laboratories. The present study compared efficacy of propofol of different laboratories and different concentrations (1 and 2%) during induction of anesthesia.* Methods*. Seventy-five scheduled surgical patients were randomly allocated into three groups. The patients of group D1 received AstraZeneca Diprivan 1% (Osaka, Japan) at a rate of 40 mg kg^−1^ h^−1^. Group M1 was given 1% Maruishi (Maruishi Pharmaceutical, Osaka, Japan) and group M2 was given 2% formulation at the same rate of propofol. Achieving hypnosis was defined as failure to open their eyes in response to a verbal command and the venous blood sample was withdrawn.* Results*. The hypnotic doses of M2 were significantly larger (D1: 91.4 ± 30.9, M1: 90.7 ± 26.7, and M2: 118.4 ± 40.2 mg, resp. (mean ± SD). *p* < 0.005). Age and gender were selected as statistically significant covariates using general linear model-ANOVA. The blood concentration showed no significant difference among the groups (3.73 ± 2.34, 4.10 ± 3.04, and 4.70 ± 2.12 *μ*g mL^−1^, resp.).* Conclusion*. The required dose of propofol was different among the formulations; however, the serum concentration showed no significant difference. This trial is registered with UMIN Clinical Trial Registry: UMIN000019925.

## 1. Introduction

Propofol is a popular intravenous anesthetic [1] and is widely administered to patients not only for managing anesthesia but also for maintaining adequate level of sedation in Intensive Care Unit [2]. The pharmacodynamics of propofol was modified by plenty of physiological factors [3–6].

Propofol is a highly lipophilic agent and is distributed as micellized fluid composed of propofol and soybeans oil. Nowadays, varieties of formulations are produced from different laboratories. Calvo et al. [7] reported that the pharmacokinetics and pharmacodynamics of propofol were not equal among the formulations. The five products were studied for determining each pharmacological property; however, the concentration of propofol and the composition of soybeans oil were almost the same.

The aim of the present study was to compare the efficacy of propofol of different laboratories (differences in solvents) and concentrations (1% versus 2%) during the induction of anesthesia in clinical settings.

## 2. Materials and Methods

A prospective double-blind control study was conducted after obtaining approval from the Institutional Review Board of Hamamatsu University School of Medicine (Registration number: 20-22, Ethics Committee of Medicine). All interventional procedures, including written informed consent from the participants, conformed to the study protocols.

Seventy-five scheduled surgical patients, ASA physical states I and II without any severe cardiovascular, respiratory, liver, and renal complications, were enrolled into the study. The sample size was determined as both effect size and statistical power are 0.8. The patients received no premedication on the day of surgery.

In the operating room, standard ECG, noninvasive blood pressure, and pulse oximetry were monitored and recorded. The intravenous infusion catheter was placed on the back of hand. The participants were randomly allocated into three groups using an envelope method. After preoxygenation using face mask, the patients of group D1 received Diprivan (1%, AstraZeneca) at a rate of 40 mg kg^−1^ h^−1^ [3]. Patients of group M1 were given 1% Maruishi (Maruishi Pharmaceutical) and of group M2 were given 2% Maruishi at the same rate of propofol. The carrier fluid was infused at a rate of 300 mL h^−1^. Achieving hypnosis was defined as failure to open their eyes in response to a verbal command with light stimuli (tactile) by an anesthesiologist in charge and 3 mL of venous blood sample was simultaneously withdrawn at the cubitalis vein of upper extremities. After confirming the hypnosis, the infusion rate of propofol was reduced to 10 mg kg^−1^ h^−1^, and 0.1 to 0.2 mg of fentanyl and 5 to 10 mg of vecuronium were subsequently administered for orotracheal intubation.

The venous sample was centrifuged and the serum was stored at −40°C until analysis. The other day, the serum concentration of propofol (*C*_*p*_) was determined using an HPLC with a fluorescence detector [3, 6].

The required dose of propofol for hypnosis and *C*_*p*_ were analyzed using general linear model-ANOVA using patients' background parameters including age and gender as covariates. A *p* value less than 0.05 was considered as significant and subsequent post hoc analysis was applied using Newman-Keuls multiple comparison test. All calculations were performed using statistical software (NCSS2000, NCSS, LLC, Kaysville, UT).

## 3. Results

**Figure 1:**
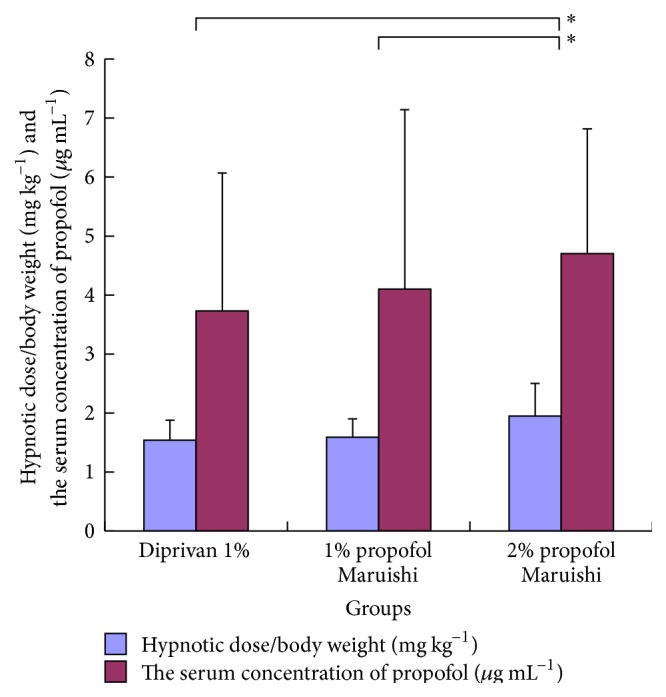
The results of the hypnotic dose of propofol and the serum concentration of propofol. The results were expressed as mean ± SD. ^*∗*^ *p* < 0.05 between the groups.

All patients completed the study. There was no significant difference in the patients' characteristics among the groups (Table 1). The hypnotic doses of groups D1 and M1 showed no significant difference (statistical power = 0.80), whereas the dose of group M2 was significantly larger (91.4 ± 30.9, 90.7 ± 26.7, and 118.4 ± 40.2, resp. (mean ± SD). *p* < 0.005, Table 1, Figure 1). Age (−0.66 ± 0.19 mg a year (regression coefficient and SE), *p* < 0.002) and gender (−28.6 ± 6.9 mg, *p* < 0.03) were selected as statistically significant covariates for the general linear model for the dose of propofol. Elderly and female patients included potential factors decreasing the required dose of propofol for achieving hypnosis in the current study population. *C*_*p*_ showed no significant difference among the groups (3.73 ± 2.34, 4.10 ± 3.04, and 4.70 ± 2.12 *μ*g mL^−1^, resp. (mean ± SD)). The age and gender were not correlative with *C*_*p*_ in all 75 patients.

## 4. Discussion

The results of current investigation demonstrated that the efficacy of propofol was distinctive not by the difference of solvents (long-chain triglycerides (Diprivan) versus long- and medium-chain triglycerides (Maruishi)) but by the difference of concentration. Higher concentration of propofol compound showed lower efficacy for achieving hypnosis. Although age and gender were selected as significant determinant factors for propofol induction dose, *C*_*p*_ was independent of the parameters.

The pharmacodynamics between blood and brain might depend on the concentration of free propofol in aqueous phase [8, 9]. There is a possibility that the differences of solvent would modify the efficacy of propofol [7, 10]. We use generic or copy formulations of propofol and the equivalency or dissimilarity among the products becomes a worrisome subject; however, the detailed information was limited [11]. The identity of the distributed formulations should be secured. If there is an apparent difference between the drugs, physicians are required to modify the dose of drugs and the timing of administration. We preliminary studied the efficacy of propofol in the institute during the induction of anesthesia [3] and reported the independent physiological factors on the hypnotic dose of propofol. The infusion method [3] was considered as showing high sensitivity, and the current study investigated a slight difference using the same manner.

There is no clear explanation for the results concerning the effect of the concentration. In the preliminary and laboratory animal experiments, we found opposite results that the diluted propofol showed greater potency [12]. In clinical settings, variability of the effect of propofol might absolutely depend on pathophysiological backgrounds [13] and the difference of formulations might be negligible. Indeed, the results of current study showed that the patient's age and gender were confirmed as significant covariates of the results. There is a possibility that the difference of efficacy of formulation might be a sequel of the interferences between these physiological factors and other unknown properties, not of the concentration of propofol.

Recently, water soluble propofol has been developed [14, 15]. Anesthesiologists will have much more options for induction of anesthesia and for maintenance of adequate sedation, and the further investigations for evaluating the properties of each formulation are absolutely required.

## Key Messages

Anesthesiologists will have much more options for intravenous anesthetics, and the further investigations for evaluating the properties of each formulation are absolutely required.

## Figures and Tables

**Table 1:** Patients' demographic data and the results.

	Diprivan 1%	1% propofol Maruishi	2% propofol Maruishi
Age (yr)	60.4 ± 18.4	60.1 ± 17.7	55.1 ± 18.6
Sex (m/f)	11/14	14/11	10/15
Body height (cm)	157 ± 8.9	161 ± 8.9	161 ± 8.2
Body weight (kg)	58.6 ± 11.3	56.7 ± 8.8	60.5 ± 11.0
Hypnotic dose (mg)	91.4 ± 30.9	90.7 ± 26.7	118.4 ± 40.2
Hypnotic dose/body weight (mg kg^−1^)	1.54 ± 0.34	1.59 ± 0.31	1.95 ± 0.55^*∗*^
The serum concentration of propofol (*µ*g mL^−1^)	3.73 ± 2.34	4.10 ± 3.04	4.7 ± 2.12

Data are expressed as mean ± SD. ^*∗*^ *p* < 0.05.
